# Anomaly Detection of GAN Industrial Image Based on Attention Feature Fusion

**DOI:** 10.3390/s23010355

**Published:** 2022-12-29

**Authors:** Lin Zhang, Yang Dai, Fuyou Fan, Chunlin He

**Affiliations:** 1 School of Computer Science, China West Normal University, Nanchong 637000, China; 2 Faculty of Artificial Intelligence and Big Data, Yibin University, Yibin 644000, China

**Keywords:** anomaly detection, attention feature fusion, generative adversarial network, image augmentation

## Abstract

As life becomes richer day by day, the requirement for quality industrial products is becoming greater and greater. Therefore, image anomaly detection on industrial products is of significant importance and has become a research hotspot. Industrial manufacturers are also gradually intellectualizing how product parts may have flaws and defects, and that industrial product image anomalies have characteristics such as category diversity, sample scarcity, and the uncertainty of change; thus, a higher requirement for image anomaly detection has arisen. For this reason, we proposed a method of industrial image anomaly detection that applies a generative adversarial network based on attention feature fusion. For the purpose of capturing richer image channel features, we added attention feature fusion based on an encoder and decoder, and through skip-connection, this performs the feature fusion for the encode and decode vectors in the same dimension. During training, we used random cut-paste image augmentation, which improved the diversity of the datasets. We displayed the results of a wide experiment, which was based on the public industrial detection MVTec dataset. The experiment illustrated that the method we proposed has a higher level AUC and the overall result was increased by 4.1%. Finally, we realized the pixel level anomaly localization of the industrial dataset, which illustrates the feasibility and effectiveness of this method

## 1. Introduction

Anomaly detection, also named as outlier detection, is a process in which actual cases are detected where there is an obvious deviation from the majority of the data [1], and then the anomalous value is found for the data distribution that is different from the body data. For the past few years, image anomaly detection has been widely applied in several research domains, such as finance detection [2], cyber security detection [3], credit card fraud detection [4], manufacturing detection [5], video surveillance detection [6], biomedicine detection [7], and so on. In the manufacturing industry, image anomaly mainly refers to the damage or threat to a product’s quality in different degrees [8]. For instance, on the surface of a finished industrial product, there may be defects, such as a scratch, erosion, a crack, and the like; some of these are obvious while some are difficult to discover. For this reason, finding inferior products and defective sites promptly not only increases product quality standards, but also localizes the operative breakdown of a machine. Therefore, industrial image anomaly detection has become especially important nowadays. As such, we took industrial image anomaly detection in the manufacturing industry as the main object to research, as it is focused on the quality detection process of finished industrial products.

Nowadays, there are three problems concerning industrial anomaly detection: Firstly, the sample is unbalanced; while the quantity of normal samples is so big that they are easy to obtain, the number of sample anomalies is so few that they are hard to collect. Secondly, to label data is difficult; and in the process of supervised learning, training the model depends on sample labeling, whereas labeling anomaly samples requires more time and labor and is less practical for the application of anomaly detection for industrial images on a large scale. Thirdly, the anomalous form appears as a diversification; anomaly means abnormal, and the size of the form’s irregularly varies randomly, there are various kinds, and the chance of the character being unknown is very strong. In this situation, the ability of automatic anomaly detection is restricted by its supervised learning, which needs a great number of known labeled samples. Schlegl et al. [7] first proposed the application of a generative adversarial network (GAN) [9] to the field of anomaly detection. Compared with other models, GAN holds its own position in the field of deep learning. It is used to explore the root cause because GAN can model complex multidimensional distribution in the real world, study the inherent law of real data, simulate data distribution, generate clearer and lifelike samples, and has displayed superior performance in various anomaly detection training tasks.

**Figure 1:**
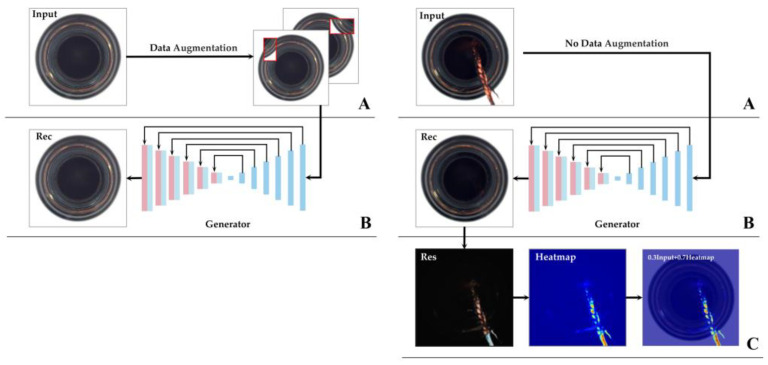
On the **left**, (**A**) is the image enhancement process and (**B**) is the image reconstruction process. Generators learn only normal image features. On the **right**, (**A**) indicates that the image has not been data enhanced. (**B**) is the reconstruction process of the test image. (**C**) represents the absolute residual between the reconstructed image and the test image, and the residual image is obtained. Finally, the thermal map is generated according to the residual image, and a 0.7 times thermal map is superimposed on the 0.3 times abnormal image to complete the abnormal location of the image.

Based on GAN, we built an industrial images anomaly detection model by using the reconstruction ability of an auto-encoder and the correlation learning ability of attention feature fusion (AFF). Firstly, for extracting most of the features, we purposefully used an encoder for extracting the feature vectors and used a decoder for reconstructing the images. Secondly, in order to extract the detail features of an image more effectively, we added the attention feature fusion mechanism to every convolution layer of the decoder and through skip-connection, we input the encode vectors and decode vectors of the same dimension into the AFF in order to capture the correlative features among the image pixels. Finally, for increasing the diversity of the limited training dataset, we added an image augmentation module. In this paper, we created an experiment with the open dataset MVTec [10] to verify the effectiveness. Figure 1 shows the detection process for our method.

Different from previous methods, we introduced an image cut-paste module to enhance the datasets, and randomly cut a certain proportion to rectangle size in the original image and then pasted it into the original image to increase the irregularity of the image in an attempt to achieve a rough simulation of anomalies. In addition, attention feature fusion is introduced to enhance the network’s attention to channel information. The multi-scale image fusion enables the network to have a stronger learning ability, to capture more detailed features of the image. To sum up, in this paper, there are three aspects of contribution as follows:

1. It proposes a novel module of an encoder–decoder GAN based on attention feature fusion, which can detect anomaly images accurately while never depending on an anomaly sample.
2. We made an attention feature fusion for the corresponding convolutional layers of both the encoder and decoder, so as to retain the channel features of different dimensions. In addition, we added extra image augmentation to simulate an anomaly for the purpose of dataset enhancement.
3. Compared with the experimental results of other similar modules, it is verified that, in the aspect of anomaly classification, our method has achieved superior performance.

The rest of this paper is organized as follows: The second section introduces the present related works and the existing research state of image anomaly detection. The third section introduces the network construction, object function, detection method and image enhancement process we proposed. The fourth section describes the dataset we used, training detail, and the experimental results as well as the ablation experiment and comparison experiment. In the fifth section, we deduce a conclusion from the experiment.

## 2. Related Work

In recent years, as deep learning arises rapidly, anomaly detection based on GAN has become a research hotspot and related applications have become more and more extensive. In 2017, based on the deep adversarial network Schlegl et al. [7] proposed, AnoGAN, which was the initial case of GAN, was used in the field of anomaly detection. The main thought behind AnoGAN is that, through a convolution neural network, a priori distribution for generating an image is input, and then the generated image and real image are input into a discriminator to be classified. Finally, the anomaly value is determined according to the residue between the generated image and the real image. However, there is a defect of low computational efficiency. To improve the training speed, Schlegl et al. [11] proposed f-AnoGAN, which rapidly mapped a picture to a certain point in hidden space. Then, it detects an anomaly by means of WGAN [12]. This model performs excellently for its variability in capturing normal samples in a smooth expression way. GANomaly [13] introduced a type of encoder-decoder-encoder network layout into GAN, which primarily compresses and maps an image into a latent feature vector and reconstructs the image, and then uses an auxiliary encoder to map the generated images into a latent expression. Under the condition of no negative case, this method achieves anomaly detection, but it cannot reconstruct the complex multidimensional data of realities well. Inspired by U-net [14], Skip-GANomaly [15] added skip-connection between corresponding convolutional layers. This skip-connection provides essential help for directing information transmission between convolutional layers. OGNet [16] used two auto-encoders as generators for high quality and low quality reconstruction, respectively, and transferred the function of the discriminator from discriminating between true and false to discriminating between high quality or poor quality images. Additionally, its author also proposed a pseudo-anomaly module for artificially constructing a false anomaly example. Bergmann et al. [17] proposed a frame of unsupervised anomaly detection on the basis of teacher-student learning, the local descriptor of the pre-trained teacher network can act as the substituted label of student sets. In addition, this paper proposed a score function based on student forecasting variance and regression error, which is beneficial to more precisely segment an image anomaly.

CutPaste [18] is the method used to enhance an image; the main thought here is that an anomaly sample is constructed in the range of the image by randomly cutting and then randomly pasting it to the image. The simulated anomaly sample is then used in motivating the model to reconstruct a normal sample well. The literature [18] proposed a high-performance image anomaly detection model that does not depend on anomaly data; it learns the features of normal images with a self-supervised model, and then constructs a single discriminator on the basis of the learned features. Fei et al. [19] transferred the images construction task to a restoration task, and proposed a simple and efficient attribute erase module (AEM) as well as an attribute restoration network (ARNet). The former can delete and compact semantics to represent the attributes of correlative color and direction. The latter can efficiently extract an image’s semantic features. In the test phase, in theory, the attribute restoration value of anomaly images should be very big. Li et al. [20] proposed an anomaly detection method based on dual attention and consistency loss; the multiple scale channel attention and pixel attention are jointly used in the generative network based on an auto-encoder. Furthermore, pixel consistency, construction consistency and gradient consistency are added to the object function in order to enhance detail information retention. For solving an unbalanced sample, Tang et al. [21] proposed DAGAN, within which the frame of skip-connection and dual auto-encoder displayed very strong reconstruction ability and stability. Likewise, Wang et al. [22] used the frame of an encoder-decoder-encoder to reconstruct images; here, the peculiar point is that the anomaly localization needs to pass through the three phases of pixel level localization, an area level localization and the fusion result. In the first phase, the localization is accomplished by the absolute value residuals of the original images and the reconstructed images. In the second phase, it proposed that the area localization is accomplished by using a local difference analysis (LDA) module. Lastly, the final segment results are obtained by the strategy of fusing the residual images with the mask images. Chen et al. [23] proposed a network constructed from a dual generator–discriminator on the basis of an encoder-decoder-encoder, which is used for improving the accuracy of anomaly detection through GAN learning, dynamically and reciprocally, via its normal distribution and marginal distribution; it also defines the optimized anomaly scores. By combining BiGAN [24] with an auto-encoder, CBiGAN [25] introduced two consistency constraints, which respectively, keeps the latent feature and spacious consistency of each image to retain the reconstruction accuracy of the model. From the above, we can see that anomaly detection on the surface of an industrial product is an indispensable part of intellectual production and possesses strong practicability and realistic significance.

As far as the above methods are concerned, the proposed method is not completely independent of normal samples, and there are some problems, such as the large amount of computing resources required, unstable reconstruction ability, and low detection accuracy. In order to overcome these shortcomings, we proposed a GAN anomaly detection method based on attention feature fusion, which is more accurate, more stable and does not depend on abnormal samples, combined with the high dimensional and complex features of detection images. Its more precise reconstruction capability improves the anomaly detection performance significantly.

## 3. Propoosed Method

### 3.1. Network Architecture

#### 3.1.1. Generative Network

**Figure 2:**
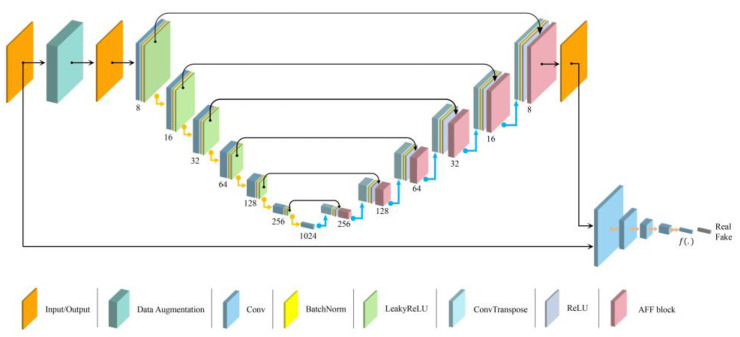
The architecture of the network used in our method.

We proposed the network architecture shown in Figure 2. Inspiration was gained from Skip-GANomaly [15] and U-net [14] and our generator employs an encoder–decoder frame, and via skip-connection, simultaneously inputs the encoding feature vectors and decoding feature vectors in the same dimension to the AFF. Encoder $G_{E}$ is mainly composed of a convolutional layer and a batch normalization [26] layer. Decoder $G_{D}$ is mainly composed of a transposed convolutional layer and a batch normalization layer. The input image passes through the decoder and vector z is obtained, then z is decoded so as to get the reconstructed image $x^{\prime }$. This is a feature extraction process; the encoding and decoding can be represented as:

(1) $$z=f_{En}\left(x,\theta_{En}\right)$$

(2) $$x^{\prime }=f_{De}\left(z,\theta_{Dn}\right)$$

where $f_{En}$ denotes the encoding function, $f_{De}$ denotes the decoding function, x is the input image, z is the latent vector, $\theta_{En}$ and $\theta_{Dn}$ denote, respectively, the parameters of the encoder and decoder, $x^{\prime }$ is the reconstructed image.

#### 3.1.2. Discrimination Network

The discrimination network, the architecture of which is identified with the discriminator of DCGAN [27], is composed of a convolutional layer, a batch normalization layer and an activation function. Besides, Sigmoid is used on the last layer, and a leaky rectified linear unit (ReLU) is used in all the other layers as an activation function. Its main function is to discriminate between the true and false image x and $G\left(x\right)$, and learns the intrinsic laws of the image data and then outputs a scalar value of 0–1. Meanwhile, the discriminator and generator alternately create adversarial training to improve the performance, respectively, in the hope of reaching the Nash Equilibrium [9]. Moreover, the discriminator can be used as a classifier as well as a feature extractor.

#### 3.1.3. Attention Feature Fusion

**Figure 3:**
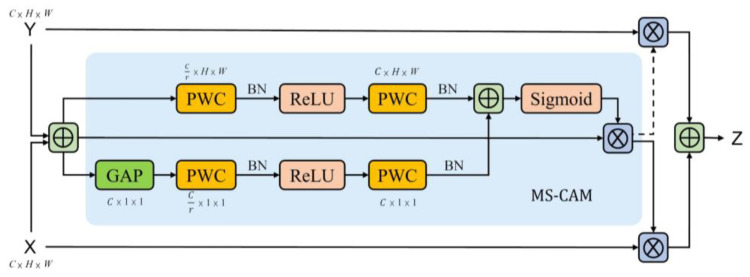
Attention feature fusion: the blue area represents the multiple scale channel attention module, ⊕ denotes the broadcasting addition, and ⊗ denotes the element-wise multiplication.

In order to improve the effect of the reconstruction and capture richer image channel features of different convolutional layers, and with inspiration from Dai et al. [28], we added the attention feature fusion (AFF), the concrete network architecture of which is shown in Figure 3.

AFF is constructed on the basis of a multiple scale channel attention module (MS-CAM). Firstly, X and Y are input for initial feature integrating, which yields F. Then, F is input to MS-CAM to intensify the attention of the network on image channel information. Finally, the result of intensification is, respectively, performed as an element-wise multiplication with the X and Y element, the summation of which is the fusion feature *Z*.

(3) F=X⊕Y

(4) $$Z=X⊗M\left(F\right)+Y⊗\left(1-M\left(F\right)\right)$$

where, X and Y represent the object of fusion, F is the result of the initial integration, which is also called the broadcasting addition, Z represents the fusion feature, M represents MS-CAM, ⊕ denotes the broadcasting addition, and ⊗ denotes the element-wise multiplication.

MS-CAM extracts channel information from the feature map and includes two parts: the global and the local. For the global feature of channel attention, the global average pooling operation should be performed for the input feature map F firstly. Then, it is convoluted point-wise by the kernel size of $\frac{C}{r}*1*1$, again, it is convoluted point-wise by the kernel size of C∗1∗1 after the operations of batch normalization and ReLU activation function. Finally, $F_{1}$ is obtained after the treatment of batch normalization. The process is shown as follows:

(5) $$F_{1}=BN\left(PC\left(ReLU\left(BN\left(PC\left(GAP\left(F\right)\right)\right)\right)\right)\right)$$

where GAP represents global average pooling, PC represents point-wise convolution, BN represents batch normalization, and ReLU is activation function.

In contrast, for the local feature, there is not a global average pooling operation on the extracting channel attention feature, and the two point-wise convolutional kernel sizes, respectively, are $\frac{C}{r}*H*W$ and C∗H∗W, through which calculation $F_{2}$ is obtained. The process is shown as follows:

(6) $$F_{2}=BN\left(PC\left(ReLU\left(BN\left(PC\left(F\right)\right)\right)\right)\right)$$

Then, combining the channel attention broadcasting addition of the global feature and local feature, and using the Sigmoid activation function, the attention map $F^{\prime }$ is obtained.

(7) $$F^{\prime }=Sigmoid\left(F_{1}⊕F_{2}\right)$$

Finally, map F is input and is multiplied element-wise by the attention feature map, whereby the MS-CAM output feature $M\left(F\right)$ is obtained.

(8) $$M\left(F\right)=F⊗F^{\prime }$$

Attention feature fusion can intensify association learning of the image feature, and can extract the association features between pixels to the fullest, so as to make the representation data richer. Therefore, the overall quality of the generated image will be improved. We take the encode feature vector and the decode feature vector in the same dimension as X and Y to be input into the AFF of the identical dimension, and through this calculation, obtain the fusion feature.

### 3.2. Data Augmentation

**Figure 4:**
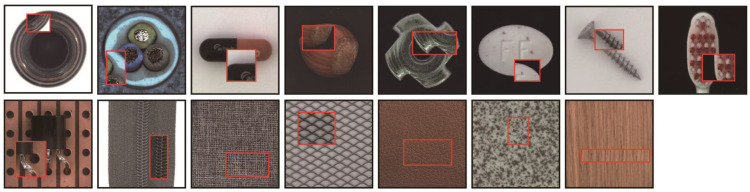
Examples of the image augmentation effect. This method randomly cuts a small rectangular area (the red rectangular boxes) and pastes these into random positions.

In order to prevent model over fitting, we performed an image augmentation for the training dataset. Nowadays, image augmentation is an efficient method of enriching the diversity of a dataset, and the methods of augmentation are varied. For resolving the problem of an error fail concerning the reconstruction at the pixel level, RIAD [29] proposed a method, whereby, by randomly deleting part of the image and replacing the lost information with content that is reasonable in semantics, image restoration and reconstruction are fulfilled. In consequence, RIAD transferred the question of image anomaly detection to image restoration and reconstruction. The augmentation method of random erasing [30] has been widely applied, within which, the mask area length and width, and the pixel substitution values, are random. In operations such as classification, detection and masked face recognition, this method can mask the image in varying degrees to obtain a robust effect. Inspired by CutPaste [18], we added an image augmentation module to the model. Shown in Figure 4, we randomly cut rectangular areas, based on an area ratio from 0.02 to 0.15, and then randomly pasted these to the original images to intensify the irregularity of the image’s content for the purpose of achieving the effect of sketchily simulating an actual anomaly. Concerning the pasted contents, the attributes of size, rotation angle and color dithering can be adjusted. The size of our augmentation module can be chosen randomly, as can the part of the texture categories of the dataset and applied color dithering treatment. Lastly, the augmented data was input into the generator for training.

### 3.3. Training Objectives

During the training, we first input a normal sample to the image augmentation module, where it was processed to obtain an artificially construed false anomaly image. Then, this image was transmitted to encoder $G_{E}$; as a consequence, the compressed feature vector z was obtained. After being reconstructed through decoder $G_{D}$, the generated image was obtained. At that very moment, the network simultaneously input the generated image and the original image to the discriminator to be discriminated, and the discriminator output a scale value of 0–1. As we only trained a normal image, the generator only learnt the detail features of a normal image. When a normal image is input into the model, in theory, there should be a similarity to a certain degree. When an anomaly image is input into the model, the generator cannot reconstruct the anomaly. In other words, the generator should possess the ability to reconstruct part of an anomaly into a normal feature. When the model calculates the content loss of a generated image and an anomaly image, the theoretical value of the loss should be big.

To improve the training network, and to meet different requirements, we employed three loss functions; and by combining these with weights, we obtained an overall objective function.

**Adversarial Loss:** In order to enable the model to reconstruct an input sample to the greatest degree, through adversarial training, we made the discriminator lack the ability to discriminate true or false from two kinds of images, with the aim of making the model generate more lifelike samples. By minimizing adversarial loss, generator G should learn the basic features of a normal image. Its loss function is defined as follows:

(9) $$L_{adv}=\frac{1}{N}\sum_{x\sim p_{x}}\left[logD\left(x\right)\right]+\frac{1}{N}\sum_{x\sim p_{x}}\left[log\left(1-D\left(G\left(x\right)\right)\right)\right]$$

**Reconstruction Loss:** Adversarial loss can only fit with the approximate distribution of the input sample. In order to allow the generated image to more closely reach an identity with normal image details, and to capture richer image features and content information, we used the *L*_1_ distance between the input image *x* and the generated image *G*(*x*) as the reconstruction loss to promote the approximation to infinity. Its loss function is defined as follows:

(10) $$L_{rec}=\frac{1}{N}\underset{x\sim p_{x}}{\sum }{\left|\left|x-G\left(x\right)\right|\right|}_{1}$$

**Latent Loss:** In order to ensure that the network is able to generate a reasonable latent representation, we used the last convolutional layer output of the discriminator to extract the latent representation of the generated image and normal image. We used the *L*_2_ distance from the input to the reconstructed image as the latent loss. Its loss function is defined as follows:

(11) $$L_{lat}=\frac{1}{N}\sum_{x\sim p_{x}}{\left|\left|f\left(x\right)-f\left(G\left(x\right)\right)\right|\right|}_{2}$$

where $f\left(\cdot \right)$ represents discriminator mapping from the input to the output of the last convolutional layer.

To sum up the above, the overall loss of GAN can be expressed as:

(12) $$L_{total}=W_{adv}L_{adv}+W_{rec}L_{rec}+W_{lat}L_{lat}$$

where $W_{adv},\;W_{rec}$ and $W_{lat}$, respectively, are the weights of the three corresponding loss functions.

### 3.4. Anomaly Detection

In order to discriminate an anomaly better, having referred to literature [13], we defined the anomaly score. When the detection sample is a normal image, $A\left(x\right)$ should be less and close to 0. When the detection sample is an anomaly image, $A\left(x\right)$ should be very big. For the given anomaly image, its anomaly score can be calculated by:

(13) $$A\left(x\right)=\gamma L_{rec}+\left(1-\gamma \right)L_{lat}$$

where $L_{rec}$ and $L_{lat}$, respectively, represent the value of reconstruction loss and latent loss. $L_{rec}$ measures the similarity in content of the two images, $L_{lat}$ measures the similarity in the latent feature of two images. γ denotes the weight parameter in the range of [0, 1], which represents the importance of $L_{rec}$.

The anomaly value of the detection image can be calculated by formula (13). For measuring the overall anomaly matter, having referred to literature [13] we proposed the method that reduces and magnifies the anomaly score feature into the probability range of [0, 1]; the bigger its value is, the higher the probability of an anomaly arising in the detection image. Ideally, a normal sample $A^{\prime }\left(x\right)=0$, and an anomaly sample $A^{\prime }\left(x\right)=1$. The final anomaly score can be represented as:

(14) $$A^{\prime }\left(x\right)=\frac{S\left(x\right)-min\left(S\right)}{max\left(S\right)-min\left(S\right)}$$

## 4. Experiment

### 4.1. Datasets

The importance of choosing a suitable dataset for experimental research is obvious to everyone, and different datasets are chosen according to the requirements of each different research objective and application. We chose MVTec [10] to test the model’s performance, which is an anomaly detection benchmark dataset for simulating real industrial environments, and which possesses a certain challenge meaning and a very strong reference value.

**Figure 5:**
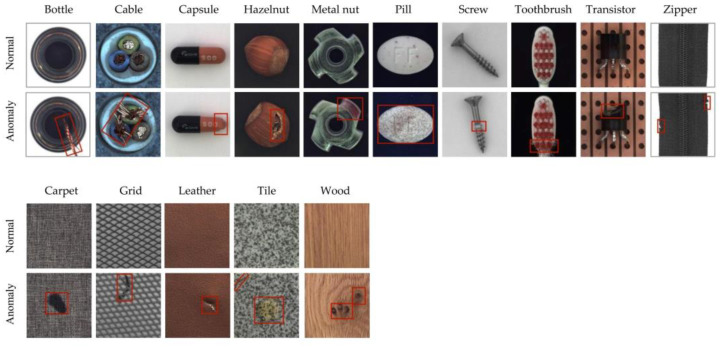
Normal samples and samples with surface defects in the MVTec dataset. The area in the red box contains the surface defect of each product.

As shown in Figure 5, the MVTec contains 15 categories, which includes 5 categories of texture (carpet, grid, leather, tile and wood) and 10 categories of object (zipper, pill, transistor, capsule, bottle, toothbrush, metal nut, hazelnut, cable and screw). The dataset contains 5354 color images of industrial products with high resolution, which ranges from 700 × 700 to 1024 × 1024. Within it, 3629 images are used for training and verification, and 1725 images are used for testing. The detailed information is shown in Table 1. The training set contains normal images, and the test set contains both normal images and anomaly images. There are 73 abnormal forms, such as damage, contamination, twists, scratches, dents, and so on. In addition, it provides anomaly images with pixel scale annotation, which is very helpful for anomaly localization.

### 4.2. Training Details

To ensure the training went smoothly, we used Adam [31] as the optimizer, with the initial learning rate set to 0.0002, and the momentum parameter set to $\beta_{1}$ = 0.5, $\beta_{2}$ = 0.999. Concerning the hyperparameter of loss function, we chose $W_{adv}$ = 1, $W_{rec}$ = 40, $W_{lat}$ = 1. The number of training epochs was set as 400, and the batch size set to 64. In this work, a PyTorch deep learning frame was used, and the hardware environment was an Intel i7-12700 and Nvidia3090 24 GB GPU.

For the training, we resized the input image of texture category and object category to 256 × 256, and randomly cut a rectangular box into an image, then randomly pasted this to the input image.

### 4.3. Evaluation

To evaluate the performance of the anomaly detection on industrial images, we employed a receiver operating characteristic area under curve (AUC) [32] as a measure standard to illustrate the discrimination effect, and for the discriminator to judge between superior and inferior. The AUC value was within the range of 0 to 1, the bigger the value was, the better effect is illustrated. If the AUC = 0.5, the predicted effect from the model is equivalent to a random guess.

### 4.4. Experimental Results

#### 4.4.1. Anomaly Classification

**Figure 6:**
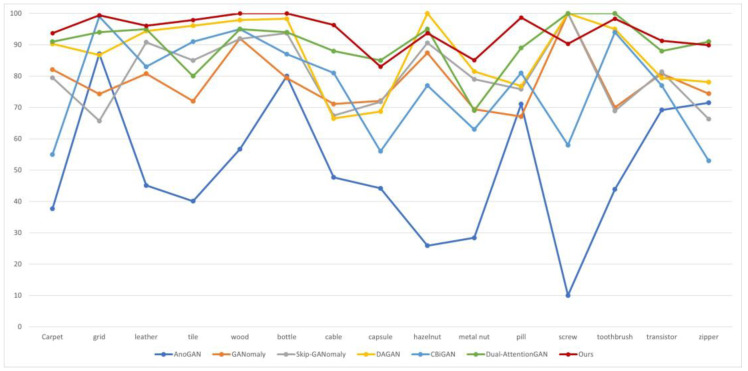
Visualization of the AUC values of the method we proposed and the other six methods.

The results of the anomaly detection based on AFF are shown in Table 2. We compared these with AnoGAN [7], GANomaly [13], Skip-GANomaly [15], DAGAN [21], CBiGAN [25] and Dual-AttentionGAN [20]. Among those, the AUC data in AnoGAN, GANomaly, Skip-GANomaly and DAGAN are taken from the literature [21]. From Table 2 and Figure 6, it can be seen that with respect to the MVTec dataset, AnoGAN performs the worst, although it is first to apply the adversarial concept to the detection of fundus oculi pathology. In contrast, on most of the categories, our method achieved the most advanced AUC, and the AUC average value reached an optimum, despite it performing poorly on a few categories. Compared with dual-attention GAN, which possesses consistency, with respect to the average AUC of the detection effect, our model increased it by 4.1 percentage points, including that of the texture category, which increased by 6.4 percentage points, and the object category, which increased by 2.8 percentage points. With comparison to the algorithm Skip-GANomaly with skip-connection, our method increased it by 13.8 percentage points. Additionally, from Table 2 it can be seen that on the texture category dataset, our method performs better, with the average AUC value reaching 97.4%. Of note, concerning the categories of wood and bottle, it reached 100%.

#### 4.4.2. Anomaly Localization

**Figure 7:**
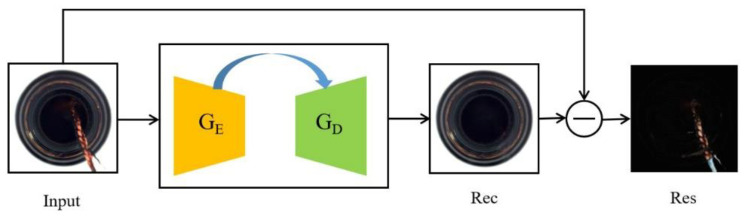
The algorithm frame diagram of the encode–decode with a skip-connection.

In industrial production, anomaly localization has a very practical guiding significance for actual production. Concerning anomaly localization, we allowed the generated images and input images to be made into absolute value residuals at the pixel level. Because the generator has only learned the features of normal samples, in theory, the local anomaly cannot be reconstructed, and the residuals of the generated images and input images are very big. As a result, the localization is achieved. As shown in Figure 7, we accomplished image reconstruction by using the encode–decode architecture frame with a skip-connection. After the anomaly input image passes through $G_{E}$ and $G_{D}$, the reconstructed image is obtained; then, the input image is compared with the reconstructed image on the pixel scale, and through the difference, the absolute value between both images of the residual image is obtained. The calculation process is shown as formula (15):

(15) $$I_{res}=abs\left(I_{in}-I_{out}\right)$$

where $abs\left(\cdot \right)$ represents taking the absolute value, $I_{in}$ represents the detection image, $I_{out}$ represents the generated image, and $I_{res}$ represents the residual image.

**Figure 8:**
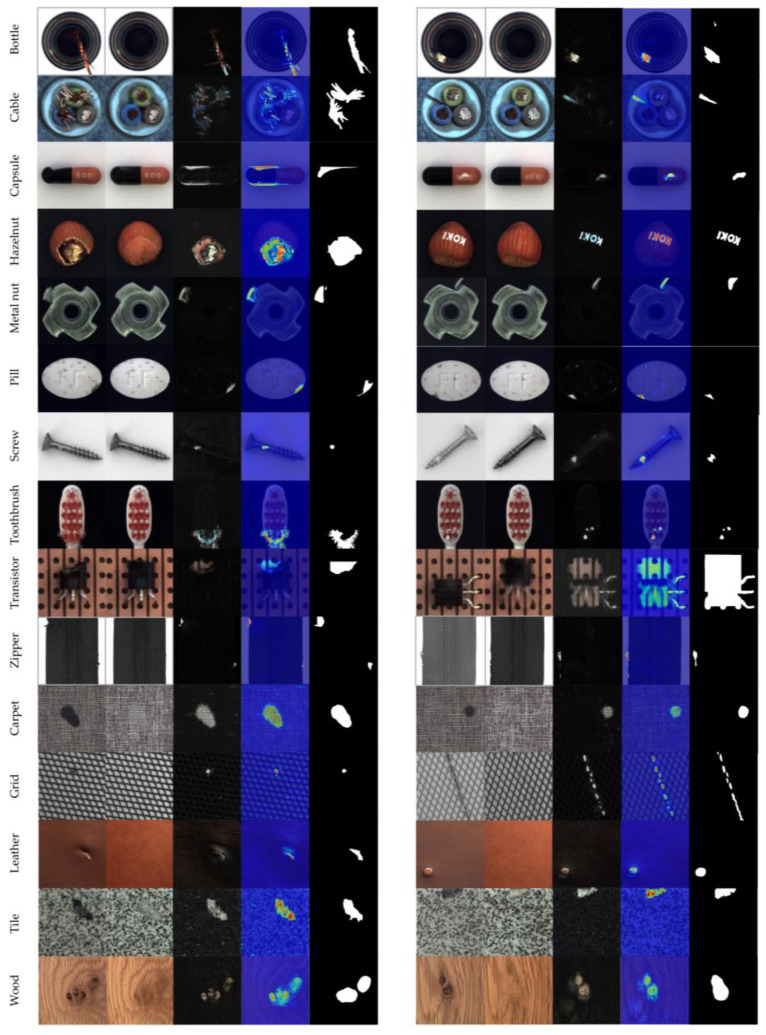
The detection results of all of the categories. From left to right: anomaly images, reconstructed images, residual image, heat map, and ground truth.

Figure 8 shows the visualization effect of the anomaly detection for 15 categories in the MVTec. In this figure, the columns respectively represent the anomaly images, reconstructed image, residual image between the former two columns, heat maps of corresponding anomaly, and the ground truth. The anomaly heat maps are obtained by superposing the pseudo color onto the original images, which are a consequence of the corresponding residual images being obtained in accordance with the reconstruction errors between the detection image and the generated image. In the anomaly heat map, the colors from blue to red represent the anomaly degree from low to high. Compared with the ground truth, it can be seen that our detection effects are similar. When the ground truth is contrasted with the anomaly heat map in Figure 8, the method we proposed can be seen to infer the anomaly area with accuracy. The higher the anomaly degree, the higher the corresponding score.

### 4.5. Ablation Studies

To evaluate the influence of single components in the model on detection performance, we used an ablation experiment. This section illustrates the effectiveness of image augmentation and AFF. We summed up the ablation result on the MVTec dataset and expressed this in Table 3. The ablation experiment mainly includes four states: State 1 represents how the generator is composed of the foundational parts of the encoder–decoder and is used to evaluate the detection performance at baseline. State 2 represents the bottom of the baseline, where an image augmentation module is added to evaluate its influence on defeat detection performance. State 3 represents how the AFF module is added to evaluate the effectiveness of the defeat detection. Finally, both of these are combined to form the model we proposed, which is State 4.

As shown in Table 3, we reported the AUC values of every sub-module on the MVTec. From Table 3, it can be seen that, as the image augmentation and AFF module are used alone for the detection task, when compared with the baseline, the performance increased by 0.5% and 4.3%, respectively. However, when united, image augmentation and AFF can markedly improve the detection effect, and consequently, the average AUC surpasses the benchmark model by 18.8 percentage points. It illustrates the effectiveness of combining the above two modules. This proves that image augmentation is helpful in enlarging data volume, and AFF is helpful in the classification detection of the auxiliary network. Comparatively, in State 4, the model we proposed performs excellently and the average AUC reaches 97.4%, especially in the wood category, where it reaches 100%. In addition, among the object categories, there are eight categories that increase, and they, as a whole, perform well with an average AUC up to 92.7%.

### 4.6. Comparative Experiment

To evaluate the influence of unitedly using the different components on detection performance, we used a comparative experiment. In this this section, we illustrate the superiority of the cut-paste image augmentation and AFF module. We summed up the comparative result on the MVTec and this is expressed in Table 4. The comparative experiment mainly included the four combinations of random erasing with connection, random erasing with AFF, cut-paste with connection, and cut-paste with AFF. Here, random erasing means randomly choosing a rectangular area and erasing all pixels within it. Connection means tensor splicing the corresponding convolution layers of the encoder and decoder. For the operation of the attention feature fusion, refer to Section 3.1.3. For the operation of cut-paste, refer to Section 3.2.

As shown in Table 3, we reported the AUC values of the different sub-modules on the MVTec. In this table, when comparing the third and fourth column with the fifth and sixth column, it can be seen that after substituting the operation of random erasing with cut-paste, the average AUC increased by 3.8% and 4.2%, respectively. This proves that under the same conditions, the overall effect of the cut-paste data augmentation method is better than random erasing. Under the precondition of cut-paste, on average, the AUC using AFF is higher by 4.2 percentage points than a normal skip-connect. In sum, by comparing the average AUC values of the four different sub-modules mentioned above, it can be seen that the combination of using cut-paste with attention feature fusion can obtain a better effect, with an average AUC value up to 94.3%.

## 5. Conclusions

In the work that this paper has elaborated, we emphatically researched the subject of quality detection of industrial products. Through gathering together the superiority of Skip-GANomaly and AFF, we proposed a novel GAN anomaly detection method based on attention feature fusion, the key of which lies in uniting the AFF in the decoder. Through a skip-connection, it performs the feature fusion of the encode vector and decode vector in the same dimension, therefore, augmenting the attention of the generator to include global and local channel feature information. This augmented the image reconstruction ability of the generator, as well as augmenting the detection performance of the model on the conditions of sample scarcity and anomaly variation. We evaluated this model on the actual anomaly detection MVTec dataset. Compared with all of the former similar methods, on average, the AUC value of the model we proposed was higher by 4.2 percentage points than the next optimal model, and better performance was obtained. In addition, we illustrated the network effect by displaying the anomaly localization at a pixel level. Furthermore, the influence of the use of individual components and different components on the network’s detection capability was also studied. The results showed that the image enhancement and attention feature fusion modules can improve the reconstruction ability of the proposed method to some extent, which makes the method maintain a high level of AUC value.

Our method can obtain a better effect within the texture category dataset. Nevertheless, on some object categories, such as capsule, hazelnut, screw, and so on, there remains some difficulty. Consequently, we will further explore the accurate classification and localization of abnormal images within these object categories.

## Figures and Tables

**Table 1:** Detailed information of the MVTec.

	Category	Train	Test (Normal)	Test (Anomaly)	Defect Types	Image Side
Textures	Carpet	280	28	89	5	1024
	Grid	264	21	57	5	1024
	Leather	245	32	92	5	1024
	Tile	230	33	84	5	840
	Wood	247	19	60	5	1024
	Total	1266	133	382	25	-
Objects	Bottle	209	20	63	3	900
	Cable	224	58	92	8	1024
	Capsule	219	23	109	5	1000
	Hazelnut	391	40	70	4	1024
	Metal nut	220	22	93	4	700
	Pill	267	26	141	7	800
	Screw	320	41	119	5	1024
	Toothbrush	60	12	30	1	1024
	Transistor	213	60	40	4	1024
	Zipper	240	32	119	7	1024
	Total	236	334	876	48	-

**Table 2:** The AUC values of the anomaly detection task in the MVTec dataset with respect to the various models we reported. The results in bold and underlined are the best AUC results.

Category		AnoGAN	GANomaly	Skip-GANomaly	DAGAN	CBiGAN	Dual- Attention GAN	Ours
**Texture**	Carpet	37.7	82.1	79.5	90.3	55.0	91.0	**93.7**
	Grid	87.1	74.3	65.7	86.7	99.0	94.0	**99.4**
	Leather	45.1	80.8	90.8	94.4	83.0	95.0	**96.1**
	Tile	40.1	72.0	85.0	96.1	91.0	80.0	**97.9**
	Wood	56.7	92.0	91.9	97.9	95.0	95.0	**100**
	**Average**	53.3	80.2	80.2	93.1	84.6	91.0	**97.4**
**Object**	Bottle	80.0	79.4	93.7	98.3	87.0	94.0	**100**
	Cable	47.7	71.1	67.4	66.5	81.0	88.0	**96.3**
	Capsule	44.2	72.1	71.8	68.7	56.0	**85.0**	83.0
	Hazelnut	25.9	87.4	90.6	**100**	77.0	95.0	93.7
	Metal nut	28.4	69.4	79.0	81.5	63.0	69.0	**85.1**
	Pill	71.1	67.1	75.8	76.8	81.0	89.0	**98.7**
	Screw	10.0	**100**	**100**	**100**	58.0	**100**	90.3
	Toothbrush	43.9	70.0	68.9	95.0	94.0	**100**	98.3
	Transistor	69.2	80.8	81.4	79.4	77.0	88.0	**91.3**
	Zipper	71.5	74.4	66.3	78.1	53.0	**91.0**	89.9
	**Average**	49.2	77.2	79.5	84.4	72.7	89.9	**92.7**
**Average**		50.6	78.2	80.5	87.3	76.7	90.2	**94.3**

**Table 3:** The AUC values of every sub-module on the MVTec dataset. Hereby, the influence of every sub-module on the anomaly detection task is illustrated. The results in bold and underlined are the best AUC results.

Category		State1	State2	State3	State4
**Texture**	Carpet	52.1	56.0	54.3	**93.7**
	Grid	83.2	78.9	93.3	**99.4**
	Leather	64.3	70.1	64.6	**96.1**
	Tile	73.3	73.6	96.9	**97.9**
	Wood	96.4	96.0	99.7	**100**
	Average	73.9	74.9	81.8	**97.4**
**Object**	Bottle	84.7	91.9	72.4	**100**
	Cable	78.8	77.8	53.3	**96.3**
	Capsule	71.3	70.1	80.0	**83.0**
	Hazelnut	82.4	79.3	86.5	**93.7**
	Metal nut	55.6	58.0	55.2	**85.1**
	Pill	78.3	80.7	**99.7**	98.7
	Screw	67.1	70.6	**100**	90.3
	Toothbrush	94.7	86.4	93.1	**98.3**
	Transistor	80.5	78.7	82.0	**91.3**
	Zipper	69.4	71.3	66.1	**89.9**
	Average	76.3	76.5	78.8	**92.7**
**Average**		75.5	76.0	79.8	**94.3**

**Table 4:** The AUC values of different sub-modules on the MVTec dataset. The results in bold and underlined are the best AUC results.

Category		Struc1	Struc2	Struc3	Struc4
**Texture**	Carpet	91.1	89.0	84.8	**93.7**
	Grid	86.0	81.5	94.2	**99.4**
	Leather	81.8	82.6	85.8	**96.1**
	Tile	90.2	83.6	**99.3**	97.9
	Wood	93.4	98.2	98.4	**100**
	Average	88.5	87.0	92.5	**97.4**
**Object**	Bottle	93.7	89.8	99.8	**100**
	Cable	83.3	83.7	75.5	**96.3**
	Capsule	68.4	**87.2**	83.2	83.0
	Hazelnut	70.7	79.9	81.2	**93.7**
	Metal nut	71.6	71.9	73.1	**85.1**
	Pill	77.2	94.7	96.3	**98.7**
	Screw	**100**	**100**	99.3	90.3
	Toothbrush	93.1	87.8	**99.7**	98.3
	Transistor	**96.8**	77.2	89.1	91.3
	Zipper	**97.4**	85.6	91.2	89.9
	Average	85.2	85.8	88.8	**92.7**
**Average**		86.3	86.2	90.1	**94.3**

## Data Availability

Not applicable.
